# Randomised controlled trial of a brief alcohol intervention in a general hospital setting

**DOI:** 10.1186/1745-6215-14-345

**Published:** 2013-10-22

**Authors:** Celia J Shiles, Una P Canning, Sandra A Kennell-Webb, Caroline M Gunstone, E Jane Marshall, Timothy J Peters, Simon C Wessely

**Affiliations:** 1 King’s College London Medical School, Guy’s Campus, King’s College London, London SE1 1UL, UK; 2 Out-Patient Department, Maudsley Hospital, South London and Maudsley, NHS Foundation Trust, Alcohol Unit, C/O Room 40, Denmark Hill, London SE5 8AZ, UK; 3 Institute of Archaeology and Antiquity, University of Birmingham, Edgbaston, Birmingham B15 2TT, UK; 4 Institute of Psychiatry, Weston Education Centre, Cutcombe Road, London SE5 9RJ, UK

**Keywords:** Brief alcohol intervention, General hospital, Alcohol problems, Controlled clinical trial

## Abstract

**Background:**

The evidence suggests that brief alcohol-focused interventions, directed at hazardous and harmful drinkers in non-specialist settings such as primary care are effective in reducing alcohol consumption. However, there is a need for further research in the hospital setting. This is a randomised controlled trial to investigate the effectiveness of a 10-minute brief intervention amongst 'at risk’ drinkers admitted to general hospital wards. Unlike some previous trials, this trial is randomised, used blinded assessors, includes an intention-to-treat analysis, included female subjects and excluded people with alcohol dependence.

**Methods:**

A total of 250 'at risk’ drinkers admitted to King’s College Hospital were identified using the Alcohol Use Disorders Identification Test (AUDIT). Some 154 subjects entered the study and were randomly allocated to the control and intervention groups. Subjects in the control group received no advice about their drinking whilst subjects in the intervention group received 10 minutes of simple advice on reducing alcohol consumption. Recruitment took place between 1995 and 1997. The primary outcome was the AUDIT questionnaire at 12 months. Secondary outcomes were a previous week’s Drinks Diary, questionnaires (General Health Questionnaire, Alcohol Problems Questionnaire and the Severity of Alcohol Dependence Questionnaire) and laboratory blood tests (gamma glutamyl transferase, mean cell volume and haemoglobin).

**Results:**

At 3-month and 12-month follow-up, all participants were included in the intention-to-treat analysis. At both time points there was no evidence of an intervention effect that could be attributed to the brief intervention. Both the intervention and control groups had an improved AUDIT score and reduced levels of alcohol consumption as measured by a subjective Drinks Diary at 3 months which was maintained at 12 months.

**Conclusions:**

This study has added further evidence on brief interventions in the hospital setting. In contrast to the recent Cochrane review by McQueen *et al*., the results of this study do not support the effectiveness of a brief alcohol intervention in general hospital wards. However our study was underpowered and there were flaws in the statistical analyses, and these limitations temper the strength of our conclusions.

## Background

Brief interventions are widely regarded as an effective strategy to reduce alcohol consumption in hazardous and harmful drinkers [1,2]. They are brief, and typically delivered by non-specialists in non-specialist settings. The majority of studies exclude individuals with moderate to severe alcohol dependence who should be referred to specialist treatment services.

There is good evidence for the effectiveness of brief interventions in primary care. The Cochrane meta-analysis of 22 randomised control trials found that alcohol consumption was reduced in the intervention group by an average of 38 g/week more than in the control group at 12-month follow-up [1]. Therefore, National Institute for Health and Clinical Excellence (NICE) guidelines recommend brief alcohol interventions in the primary care setting [3].

The general hospital setting has also been considered as an important setting for brief interventions as hazardous and harmful drinkers are frequently admitted to hospital and may have increased levels of motivation to reduce their alcohol consumption whilst recovering from an illness [4]. However, the evidence for a brief intervention in this setting is not as strong as it is for primary care.

A systematic review by Emmen *et al*. in 2004 found the evidence to be inconclusive for brief interventions in a general hospital setting [5]. Contrastingly in 2011, McQueen *et al.*’s Cochrane meta-analysis of 14 trials on brief interventions in hospitals found that alcohol consumption was reduced at 6 and 9 months in the intervention group compared to the control group [2]. However unlike in a primary health care setting, this alcohol reduction was not maintained at 12-month follow-up. More recently, a systematic review by Mdege *et al*. suggests that multiple session brief interventions may be effective, but there was no clear benefit found for single session brief interventions [6].

These reviews demonstrate the need for further trials to add to the evidence base for brief interventions in the hospital setting. Further research is also needed to investigate whether brief interventions are effective in females and to determine their optimal content and duration.

This study is a randomised controlled trial investigating the effectiveness of a 10-minute brief intervention amongst 'at risk’ drinkers admitted to general hospital wards. It followed up participants at 3 and 12 months. Unlike some trials in the Cochrane review of brief interventions in the hospital setting, this trial is randomised, used blinded assessors, included an intention-to-treat analysis and female subjects, and excluded people with alcohol dependence [2].

Recruitment took place between 1995 and 1997 (Additional file 1). An accompanying note describes the background to the late publication of the trial.

## Method

### Protocol

Over a two-year period, consecutive acute medical admissions to one of the three general medical teams at King’s College Hospital in South London were asked (usually within the first 24 hours of admission) to complete a short (10 minute) Health and Lifestyle Questionnaire (HLQ) incorporating questions on alcohol, smoking, diet, exercise, illegal drug use and alternative medicine [7]. Patients were excluded from the screening process if they were too physically ill; showed evidence of serious neurological impairment; had a severe psychiatric condition and/or had overdosed; were of no fixed abode, or refused. Patients with multiple admissions were recorded only once. Recruitment was extended to a second medical team in June 1996 when the pool of 'new’ patients in the first medical team diminished due to re-admissions. Informed consent was obtained from each patient and full ethical approval was received from King’s College Hospital.

The Alcohol Use Disorders Identification Test (AUDIT), a 10-item questionnaire designed for the early detection of hazardous and harmful drinkers, was embedded in the alcohol section of the HLQ, which also included questions on quantity and frequency of alcohol use. In line with previous work, patients scoring eight or above on the AUDIT were identified as hazardous/harmful drinkers, and invited to participate in a randomised control trial of a brief intervention.

The AUDIT-positive patients were further assessed using validated questionnaires to determine the number of alcohol-related problems; the severity of alcohol dependence; and psychological well-being. The questionnaires used were: the Alcohol Problems Questionnaire (APQ) [8], the Severity of Alcohol Dependence Questionnaire (SADQ), [9], and the General Health Questionnaire (GHQ) [10] respectively. A Drinks Diary was used to obtain the number of units of alcohol consumed daily over the previous week (1 unit = 8 g ethanol). Medical notes were scrutinised manually to obtain the admission diagnosis and diagnoses were coded according to the International Classification of Disease (ICD-10) (WHO, [11]). Each patient was also asked to provide a blood sample for assays of serum to test gamma-glutamyl transferase (GGT) and for routine haematology indices including haemoglobin (Hb) and erythrocyte mean cell volume (MCV) by standard laboratory methods. The assessment interview took approximately 45 minutes to complete and was carried out as privately as possible on the ward.

Patients were excluded from the trial if the initial screening process or the assessment interview found that they met the ICD-10 criteria for alcohol dependence. A history of delirium, seizures, hallucinations, and current or previous treatment for alcohol problems were also exclusion criteria.

### Assignment

**Figure 1:**
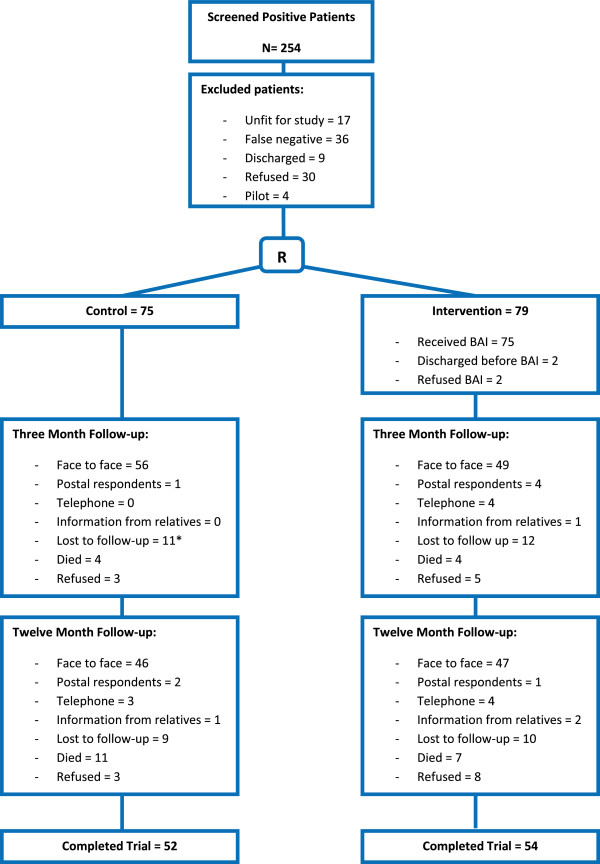
**Flow chart of patients’ randomisation and follow-up.** *Some patients lost to 3-month follow-up were contacted at 12 months. BAI, brief alcohol intervention.

At the end of the assessment interview, patients were randomly allocated to intervention and control groups by the research nurse (SKW) responsible for administering the brief intervention, using sealed envelopes. The sealed envelopes were generated by UPC and SKW in conjunction with the statistician, Richard Hooper. A total of 250 patients were eligible to join the study of whom 154 gave their consent. The study design and subject losses are shown in Figure 1.

### Intervention

Subjects in the control group received no advice about their drinking except that which they may have received from medical or nursing staff on the ward. Subjects randomised to the brief intervention group were given a 10-minute 'advice’ session by the research nurse (SKW). This session incorporated an assessment of alcohol intake, and simple advice to reduce their alcohol consumption to 21 units per week or below if male, and 14 units per week or below if female. Advice was supplemented with a number of booklets including *That’s the Limit* produced by the Health Education Authority [12]. Follow-up assessments were carried out at 3 months and 12 months by another research worker (UC) who was 'blind’ as to whether subjects had been assigned to the intervention or control groups. Subjects were contacted by telephone or invited by letter and given the choice of attending a follow-up appointment at the hospital or being seen in their own home. At the follow-up interview, the AUDIT questionnaire, and the baseline assessment interview were administered and a blood sample taken for the estimations noted above. Subjects unwilling to return for follow-up were asked to complete at least an AUDIT and a Drinks Diary, either via the telephone or by post. Subjects responding by post were offered up to £5 as an incentive.

### Masking

Documentation relating to patient assignment was logged by the research nurse (SKW) in a book kept locked in a desk. In addition, patients were given a number when entered onto the computer and were not assigned a randomisation code until the last patient had been followed up at 12 months. The research assistant (UC) who carried out the follow-up assessment arm of the study remained blind to patient allocation until the study had been completed. During the course of the follow-up interviews the 'blind’ research assistant accidently discovered how two subjects had been assigned.

### Outcome variables

The primary outcome measure was the AUDIT score at 12 months. The previous week’s Drinks Diary was taken as a secondary outcome measure, and changes in APQ, SADQ, GHQ and laboratory tests were used as subsidiary measures.

### Statistical analysis

A power calculation was based on the results of Chick *et al*.’s (1985) study in which 156 problem drinkers were assigned to a single session of counselling or routine medical care [4]. At 12-month follow-up both groups showed reduced levels of alcohol consumption, and there was no between-group difference on this variable. There was, however, an 18% difference between the groups in percentage intervention response (52% versus 34%). This percentage change may have been an artefact of the pre-intervention differences between the groups, but it represented a reasonable difference on which a power calculation could be based. To detect this difference with 80% power and 0.05 significance, 114 subjects were required for each group. In retrospect, basing the sample size on a previous study that found no difference in the primary outcome was a serious limitation. The numbers recruited were in the region of 40% of the numbers required and our study was underpowered.

Hospital data indicated that the medical team admitted 2,000 patients each year. We estimated (conservatively) that 1,000 of these could be screened (500 men and 500 women). Using data from the General Household Survey and estimates from Prof. Jonathan Chick, it was assumed that 25% of male admissions and 8% of female admissions would be identified as hazardous or harmful drinkers and it was thus estimated that 125 men and 45 women could be recruited each year into the study. The study had three years’ funding. It was, therefore, decided to screen all admissions for the first two years. Patients entering the trial in Years 1 and 2 were followed up in Years 2 and 3 respectively.

Results were analysed using CONSORT guidelines, which involved analysis both by protocol (including only those who reached the primary end point who did adhere to the protocol) and by intention-to-treat (which included all participants who reached the primary end point irrespective of whether they adhered to the treatment protocol or not).

Gender was entered into the multivariate analysis of variance (MANOVA) as an interaction effect on the two main outcome variables. Where there was a significant interaction effect, the subjects were split into male and female groups and the MANOVA repeated. The distribution of the AUDIT scores, Drinks Diary, APQ, SADQ and GHQ scores were markedly positively skewed, and to render them more amenable to statistical analysis, changes in scores were calculated that is the difference between baseline and 3 months and between 3 months and 12 months. These differences were then used in the analysis. Percentage change scores were calculated for the three blood tests. Multiple analysis of variance was used to test for time and intervention effects with gender entered as an interaction into each analysis.

## Results

### Participant flow

A total of 250 patients were eligible to enter the trial, of whom 154 gave informed consent. Of these 154 'at risk’ drinkers who entered the trial, 75 were randomised to the control group and 79 to the intervention group (Figure 1). Descriptive data at baseline for control and intervention group is shown in Table 1.

**Table 1:** Descriptive data at baseline for control and intervention groups

	**Control (n = 75)**	**Intervention (n = 79)**
**Demographic variables**	**Mean (SE) (range)**	**Mean (SE) (range)**
Age (years)	52 (1.9) (20-83)	50 (1.9) (21-78)
	**No. (%)**	**No. (%)**
Social class I-IIIn	26 (35)	26 (33)
Married	25 (33)	32 (41)
Divorced/separated	14 (19)	12 (15)
Lives with other	7 (9)	11 (14)
Single	20 (27)	20 (25)
Widowed	9 (12)	4 (5)
Lives alone	28 (37)	22 (28)
Employed	28 (37)	33 (42)

### Three-month follow-up

At 3-month follow-up, 115 (75%) of the original 154 subjects were re-assessed. Eight (5%) had died during the 3-month period leaving an available sample of 146 of whom 115 (79%) were followed up in some way: 58 from the intervention group and 57 from the control group. The unadjusted attendance rate was 73% for the intervention group and 76% from the control group. Adjusted rates, excluding those who had died, were 77% and 80% for the intervention and control groups respectively. There was no difference in baseline AUDIT scores and mean alcohol consumption between those who attended for interview at 3 months and those who did not. Of the 115 patients who were followed up, 105 (91%) successfully completed a face-to-face interview, five (4%) agreed to a telephone interview, and five (4%) returned postal questionnaires. Information on one subject (1%) was obtained from close relatives.

### Twelve-month follow-up

At 12-month follow-up 106 (69%) subjects were re-assessed. Of the original 154 subjects who entered the study, 18 (12%) had died leaving an available sample of 136, of whom 106 (78%) were followed up: 54 from the intervention group and 52 from the control group. The unadjusted follow-up rate was similar for both the intervention (68%) and the control groups (69%) and the adjusted rates were 75% and 81% respectively. There were no differences in 3-month AUDIT scores and mean alcohol consumption between those who attended for interview at 12 months and those who did not. Of the 106 patients followed up at 12 months, 93 (88%) successfully completed a face-to-face interview, seven (7%) agreed to a telephone interview, and three (3%) returned postal questionnaires. Information on three subjects (3%) was obtained from relatives.

### Analysis according to intention to treat

Means and standard deviations of outcome variables at initial assessment and follow-up for both groups are shown in Table 2. There was a reduction in the mean AUDIT score for the whole group from baseline (mean = 14) to 3-month follow-up (mean = 10) but no change was recorded between 3 and 12 months (mean = 11). This was reflected in the significant main effect of time in the analysis (F = 37.36, df 1,89, *P* <0.001). There was also a reduction in the mean unit score for weekly alcohol consumption for the whole group from baseline (mean = 51 units) to 3-month follow-up (mean = 35 units) and a further reduction in the mean APQ score for the whole group from baseline (mean = 4) to 3-month follow-up (mean = 3) and further reduction between 3 and 12 months (mean = 3) (main effect of time F = 19.68, df 1,78, *P* <0.001). No significant differences were recorded between the percentage change scores for GGT and MCV but there was a significant time effect for Hb (F = 9.81, df 1,71, *P* = 0.003). No significant intervention effects were recorded between the two groups on any of the outcome variables. There was a significant interaction effect for the weekly Drinks Diary when analysed according to 'time*gender*randomisation’ (F = 6.55, df 1,151, *P* = 0.011), but no significant intervention effects when gender was analysed separately.

**Table 2:** Data for baseline, 3- and 12-month follow-up by intention-to-treat analysis

	**Baseline**		**Three-month follow-up**		**Twelve-month follow-up**	
	**Control**	**Intervention**	**Control**	**Intervention**	**Control**	**Intervention**
	**Mean (SE) (range)**	**Mean (SE) (range)**	**Mean (SE) (Range)**	**Mean (SE) (Range)**	**Mean (SE) (Range)**	**Mean (SE) (Range)**
**Consumption**						
AUDIT + (0-40)	13 (0.6) (8-39)	15 (0.6) (8-31)	10 (0.7) (0.30)	11 (0.8) (0-33)	10 (0.7) (0-30)	11 (0.9) (0-31)
Drinks Diary	50 (4.7) (8-315)	52 (4.1) (9-200)	35 (4.3) (0-234)	36 (4.6) (0-204)	34 (4.3) (0-260)	32 (4.0) (0-124)
**Problem scores**						
APQ (0-23)	4 (0.3) (0-14)	5 (0.4) (0-18)	3 (0.3) (0-12)	3 (0.5) (0-19)	3 (0.3) (0-14)	3 (0.5) (0-19)
SADQ++ (0-12)	9 (0.3) (0-48)	9 (0.4) (0-48)	6 (0.8) (0-37)	7 (1.1) (3-48)	6 (0.8) (0-37)	7 (1.0) (0-40)
GHQ (0-12)	4 (0.4) (0-12)	3 (0.4) (0-12)	3 (0.4) (0-12)	3 (0.4) (0-12)	3 (0.4) (0-12)	3 (0.4) (0-12)
**Blood results +++**						
GGT mmol/l*	135 (43.73) (10-2867) (52)	62 (14.32) (8-1060) (35)	98.1 (17.50) (10-911)	53.3 (10.56) (8-799)	115.0 (31.77) (12-2160)	50.7 (9.52) (7-707)
MCV fl**	89.5 (1.29) (31-108) (91.3)	91.2 (0.82) (70-122) (90.5)	89.7 (1.16) (31-105)	91.2 (0.83) (74-122)	90.6 (1.28) (31-108)	91.6 (0.77) (79-122)
Hb g%***	13.6 (0.26) (5.9-17.8) (13.7)	13.4 (0.20) (8.3-17.9) (13.7)	14.1 (0.18) (9.6-17.1)	13.6 (0.20) (9.1-17.4)	13.8 (0.21) (7.4-16.9)	13.4 (0.77) (8.9-17.9)

*Excludes seven in the control group and two in the intervention group for whom no results were obtained; **excludes eight in the control group and two in the intervention group for whom no results were obtained; ***excludes six in the control group and one in the intervention group for whom no results were obtained. +Score of 8 or more = hazardous drinker; ++score greater than 30 = severe dependence; +++normal range of GGT = 5 to 55; MCV = 76 to 97; Hb 13 to 18. GGT, glutamyl transferase; Hb, haemoglobin; MCV, mean cell volume.

### Analysis according to protocol

Means and standard deviations of all the outcome variables at initial assessment and follow-up for both groups are shown in Table 3. There was reduction in mean AUDIT score for the whole group from baseline (mean = 14) to 3-month follow-up (mean = 9) but no change was recorded between 3 and 12 months (mean = 10) and this was reflected in the significant main effect of time (F = 39.96, df 1,151, *P* <0.001). A reduction in mean unit score for weekly alcohol consumption for the whole group was recorded from baseline (mean = 51 units) to 3-month follow-up (mean = 31 units) but no further reduction between 3 and 12 months (mean = 28 units) (main effect of time F = 18.11, df 1,151, *P* <0.002). There was a reduction in mean APQ score for the whole group from baseline (mean = 4) to 3-month follow-up (mean = 3) but no further reduction between 3 and 12 months (mean = 3) (main effect of time F = 18.11, df 1,151, *P* < 0.001). There were no significant differences using percentage change scores for GGT and MCV but there was a significant time effect for Hb (F = 9.17, df 1,144, *P* = 0.003). There was no significant intervention effect recorded between the two groups for any of the outcome variables. There was a significant interaction effect for weekly Drinks Diary when analysed according to 'time*gender*randomisation’ (F = 5.53, df 1,91, *P* = 0.021), but no significant intervention effects when the gender was analysed separately.

**Table 3:** Data for baseline, 3- and 12-month follow-up by protocol

	**Baseline**				**Three-month follow-up**				**Twelve-month follow-up**			
	**Control**		**Intervention**		**Control**		**Intervention**		**Control**		**Intervention**	
	**Mean (SE) (range)**	**N**	**Mean (SE) (range)**	**n**	**Mean (SE) (Range)**	**n**	**Mean (SE) (Range)**	**n**	**Mean (SE) (Range)**	**n**	**Mean (SE) (Range)**	**n**
**Consumption**												
AUDIT + (0-40)	13 (0.6) (8-39)	75	15 (0.6) (8-31)	79	9 (0.8) (0-30)	57	9 (1.0) (0-33)	58	10 (0.9) (0-30)	51	10 (1.1) (0-31)	51
Drinks Diary	50 (4.7) (8-315)	75	52 (4.1) (9-200)	79	34 (5.5) (0-234)	57	29 (5.1) (0-204)	60	34 (5.9) (0-260)	52	24 (4.5) (0-112)	53
**Problem scores**												
APQ (0-23)	4 (0.3) (0-14)	75	5 (0.4) (0-18)	79	3 (0.4) (0-12)	57	3 (0.6) (0-19)	55	3 (0.4) (0-14)	48	2 (0.5) (0-14)	40
SADQ++ (0-12)	9 (0.3) (0-48)	75	9 (0.4) (0-48)	79	6 (0.9) (0-37)	57	6 (1.4) (0-48)	55	6 (1.1) (0-37)	48	5 (1.3) (0-33)	40
GHQ (0-12)	4 (0.4) (0-12)	75	3 (0.4) (0-12)	79	3 (0.5) (0-12)	57	3 (0.5) (0-12)	54	3 (0.5) (0-260)	48	2 (0.5) (0-10)	38
**Blood results +++**												
GGT mmol/l*	135 (43.73) (10-2867) (52)	75	62 (14.32) (8-1060) (35)	79	90.1 (19.33) (10-911)	57	55.6 (16.34) (8-799)	48	3 (0.4) (0-14)	46	50.5 (16.44) (7-707)	42
MCV fl**	89.5 (1.29) (31-108) (91.3)	75	91.2 (0.82) (70-122) (90.5)	79	90.2 (0.99) (65.2-105.4)	54	90.2 (0.93) (74.1-104.8)	48	6 (1.1) (0-37)	45	90.9 (0.91) (78.6-103.4)	42
Hb g%***	13.6 (0.26) (5.9-17.8) (13.7)	75	13.4 (0.20) (8.3-17.9) (13.7)	79	14.1 (0.21) (9.6-17.1)	55	13.6 (0.24) (9.1-17.4)	48	3 (0.5) (0-12)	45	13.2 (0.28) (8.9-17.2)	42
					**Mean (SD) (Range)**		**Mean (SD) (Range)**		**Mean (SD) (Range)**		**Mean (SD) (Range)**	
**Weeks to follow-up**	N/A		N/A		15.6 (2.84) (10-22)		17.3 (6.34) (12-39)		62.1 (21.40) (47-148)		58.1 (10.58) (41-98)	

*Excludes seven in the control group and two in the intervention group for whom no results were obtained; **excludes eight in the control group and two in the intervention group for whom no results were obtained; ***excludes six in the control group and one in the intervention group for whom no results were obtained. +Score of 8 or more = hazardous drinker; ++score greater than 30 = severe dependence; +++normal range of GGT = 5 to 55; MCV = 76 to 97; Hb 13 to 18. GGT, glutamyl transferase; Hb, haemoglobin; MCV, mean cell volume.

## Discussion

The results of this study do not support the effectiveness of a 10-minute brief intervention in 'at risk’ drinkers admitted to general hospital wards. This is in contrast to the recent Cochrane meta-analysis by McQueen *et al*., but consistent with the recent systematic review by Mdege *et al*. [2,6]. However, both the intervention and control groups had an improved AUDIT score and reduced levels of alcohol consumption as measured by a subjective Drinks Diary at 3 months, which was maintained at 12 months.

There are several possible reasons why this study did not find a significant result whereas other trials have. First, brief interventions are not standardised therefore there is considerable heterogeneity across studies. The duration of the intervention in this trial was extremely short when compared with the interventions in other trials and this may have lessened the effect size. However, the review of brief interventions in primary care did not find that longer interventions were associated with a significantly greater reduction in alcohol consumption [1]. Kaner and co-authors therefore concluded that the content of the intervention may be more important than the duration of delivery.

A further explanation for the insignificant result may be the use of a single session brief intervention in this trial. A recent systematic review suggests that multiple session brief interventions may be effective, but no clear benefit was found for single session brief interventions [6].

The content of the intervention in this trial was simple advice and leaflets and this may have contributed to the insignificant result. Other studies have typically used motivational interviewing techniques [1,2]. However, further research into the most effective content of brief interventions is needed before any firm conclusions can be drawn [13]. It might be the case that different types of intervention are more suitable for different patients. For example patients who are not ready to change may benefit more from motivational interviewing whereas patients who are ready to change may benefit more from advice on how to reduce alcohol consumption [14]. A 'readiness to change’ assessment was not included in the trial so we were unable to test this.

Another possible reason for the insignificant result in this study is the size and composition of the sample. The power calculation was based on Chick *et al*.’s study [4] and this was a limitation because that study found no difference in the primary outcome and was probably underpowered. The numbers recruited in this study were, therefore, in the region of 40% of the numbers required (that is 184 subjects were needed in each group) and the trial was underpowered.

For a randomised trial the analysis undertaken in this study was flawed. The primary outcome was skewed and we used change scores (the difference between baseline and 3 months and the difference between 3 months and 12 months) as the outcome, as these appeared to be normally distributed. Furthermore, taking the AUDIT score as the single outcome, the main influence on this score at 12 months would have been the AUDIT score at baseline. Even if we used changes in AUDIT scores between baseline and 12 months, we should have adjusted the analysis for this major confounding factor by including it in the analysis as a covariate. The appropriate method for analysis would have been to employ a linear regression approach, adjusting for baseline AUDIT score to establish the mean difference between the groups in AUDIT score at month 12 and the 95% confidence intervals.

Furthermore, the inclusion of women in the trial may have diluted the effect. Four of the trials in the Cochrane meta-analysis by McQueen *et al*. included men only [2] and the primary care setting review found a significant difference for men but not for women [1]. Another difference between the sample in this study and the Cochrane review is that our trial did not include alcohol-dependent individuals whereas six of the trials in the Cochrane meta-analysis did. This should increase the likelihood of a significant result as brief interventions are thought to be effective for harmful drinking [1,2].

This trial was rigorously conducted in comparison to some of the trials included in the Cochrane meta-analysis by McQueen *et al*. It was a randomised control trial whereas half of the trials included in the meta-analysis were not randomised [2]. Furthermore, the outcome assessors were blinded, which was not the case in three of the studies included in the meta-analysis.

Like many other trials on brief interventions, this study found a reduction in alcohol misuse in both the intervention and control groups. It has been suggested that this may be due to the initial assessment process, which in this trial was of 45 minutes duration and consisted of four questionnaires on alcohol use and psychological well-being [1,2]. This could only be confirmed, however, by recruiting a parallel control group that had no initial assessment at all, probably via a Zelen design [15]. The improvement in both groups could also be explained by regression to the mean and the impact of being admitted to hospital. Furthermore, King’s College Hospital had a strong interest in alcohol misuse both amongst the psychiatrists and physicians and thus normal practice might have caused both groups to improve.

## Conclusions

In conclusion, this study has added further to the evidence base on brief interventions in the hospital setting. Unlike some previous trials included in the recent Cochrane review by McQueen *et al*., this trial is randomised, used blinded assessors, included an intention-to-treat analysis, included female subjects and excluded people who are alcohol dependent [2].

The results of this study do not support the effectiveness of a brief intervention comprising of 10 minutes 'advice’ in 'at risk’ drinkers admitted to general hospital wards. This is in contrast to the recent Cochrane meta-analysis result found by McQueen *et al*. [2]. However, our study was underpowered and there were flaws in the statistical analyses, and these limitations temper the strength of our conclusions.

Like many other trials, this trial found an improvement in both the intervention and control groups for AUDIT scores and a subjective Drinks Diary measure of alcohol consumption. This may be due to the initial assessment process, regression to the mean or the impact of being admitted to hospital.

Further research into brief interventions is needed to clarify their effectiveness in the hospital setting. In addition, further investigation is required to determine whether the intervention is effective in women and the optimum duration and content of the intervention.

## Abbreviations

APQ: Alcohol problems questionnaire; AUDIT: Alcohol use disorders identification test; GGT: Gamma glutamyl transferase; GHQ: General health questionnaire; Hb: Haemoglobin; HLQ: Health and lifestyle questionnaire; MANOVA: Multivariate analysis of variance; MCV: Mean cell volume; NICE: National institute of clinical excellence; SADQ: Severity of dependence questionnaire.

## Competing interests

The authors declare that they have no competing interests.

## Authors’ contributions

CJS drafted the introduction, discussion and conclusions. UPC, SAK, CMG, EJM, TJP and SCW conducted the trial and drafted the manuscript. All authors read and approved the final manuscript.

## Authors’ information

CJS is a fourth year King’s College London medical student. EJM is a Consultant Psychiatrist and Senior Lecturer in Addictions, South London and Maudsley NHS Foundation Trust. SCW is the Vice Dean, Academic Psychiatry, Teaching and Training: Institute of Psychiatry.

## Editor’s note

It is our policy that the trials described in articles in the journal must have been registered. The trial reported in this article was completed some years before registration was introduced so we have waived the requirement.

## Supplementary Material

Additional file 1 A note from the authors, explaining the history of this trial. Click here for file
