# Supplementary material for: Randomised controlled trial of a brief alcohol intervention in a general hospital setting
Source: Trials. 2013 Oct 22;14:345. doi: 10.1186/1745-6215-14-345 (PMC4016505; doi:10.1186/1745-6215-14-345)
Supplement: Additional file 1 — A note from the authors, explaining the history of this trial. [file 1745-6215-14-345-S1.docx]

**A note from the authors**

The reader is sure to note that this trial was conducted between 1995 and 1997. So some explanation is needed.

The trial was conducted at King’s College Hospital with the support of a grant from the now defunct South East London Health Authority. It was concluded on time and within budget. A manuscript was prepared and submitted to the BMJ. The paper was rejected with comments from the referees. And now the problems start.

By this time the research team had split up. The grant was finished, so the project co-ordinator who had prepared the first draft (UC), moved to another post outside research, as did the two research assistants. JM started to revise the manuscript following the suggestions of the BMJ referees. However, JM moved into a full time clinical post, and was exceptionally committed to new service developments. SW and TP meanwhile were also involved in new academic projects, whilst SW was also very busy setting up two new academic units. Contact was lost with UC. SW tried a few times to resurrect the trial, but was hampered by only having an out of date paper version of the manuscript. Gradually the memory of the trial started to fade away.

And then SW met Sir Iain Chalmers and Prof Altman at the 2011 BMJ Xmas party. The conversation turned to the topical issue of unpublished trials. SW mentioned that he was feeling guilty about sitting on just such a trial for over a decade. He was naturally berated by Sir Iain, but at the same time Prof Altman mentioned a new journal that was committed to publication of trials, even old ones.

And so SW decided to have another go at getting the trial to publication. Although we were unable to locate the data set, JM managed after a considerable effort to find the electronic copy of the manuscript, which included the revisions suggested by the BMJ. SW meanwhile had suggested a Special Study Component (SSC) for a KCL medical student, consisting of a project on non publication of trials, linked to resurrecting and updating an example of the genre. CS now joined the team, updated the introduction and discussion, and carried out a meta-analysis to see how our trial would influence knowledge. And so 19 years after we wrote the grant, and 17 years after we finished the study, finally we hope that this RCT will now see the light of day.

We feel that there is a message in this. It is easy to claim that non publication of trials may be result of deliberate decisions, such as a wish not to publicise negative results, and it does seem likely that this does indeed happen. But probably more common is a more understandable and human sequence of events. A first attempt at publication fails. Teams break up. Some leave academic life and start families. Others move into busy clinical posts, or take on new demanding projects. Data sets can be corrupted, and manuscripts go missing. And time marches on.

But what we hope we also show is that all is not lost. The most important thing is that we have finally got our principal results into the public domain. Of course this is not ideal, but perhaps the most important thing is that the data from this trial is now in the public domain and in a form that enables it to be available to contribute to the next systematic review and meta analysis in a field that continues to be a very important public health problem, and for which we are still short of definitive RCT evidence.

With the benefit of hindsight and the assistance of the contemporary referees in 2013 we accept that the trial was underpowered.  We also accept that there are now better statistical techniques available to analyse the data, but sadly these are denied us.  However, we also feel that even after over a decade, this study makes an important contribution to an ongoing debate, and should at least be available to readers and to the authors of future systematic reviews and/or meta-analyses. Coincidently a BMJ article by Doshi et al has recently called for other unpublished trials to be published within the next year (1).

**Reference**

1. Doshi P, Dickersin K, Healy D, Vedula S, Jefferson T: **Restoring invisible and abandoned trials: a call for people to publish the findings**. *BMJ* 2013;346:f2865
